# Planning for the Unexpected and Unintended Effects of mHealth Interventions: Systematic Review

**DOI:** 10.2196/68909

**Published:** 2025-08-07

**Authors:** Weidan Cao, Xiaohui Cao, Andrew David Sutherland

**Affiliations:** 1 Department of Biomedical Informatics, College of Medicine, The Ohio State University, 1800 Cannon Drive, 250 Lincoln Tower, Columbus, OH, 43210, United States, 1 6146853181; 2 School of Journalism and Mass Communication, University of Wisconsin-Madison, Madison, WI, United States; 3 Edward R. Murrow College of Communication, Washington State University, Pullman, WA, United States

**Keywords:** unintended effects, mHealth interventions, systematic review, ecological typology, mobile phone

## Abstract

**Background:**

Mobile health (mHealth) interventions can produce both intended and unintended effects. Examining these unintended effects helps create a more complete and objective understanding of mHealth interventions and can reduce potential harm to participants. Existing studies on the unintended effects, which were published several years ago, tend to have either a general focus on health IT or a specific focus on health care providers, thereby excluding other key stakeholders (eg, patients and community health workers). Additionally, these studies did not systematically outline the causes of the unintended effects or strategies for their prevention.

**Objective:**

To address this gap, this systematic review, guided by the ecological framework, aims to systematically identify the unintended effects of mHealth interventions, create a typology for them, investigate the reasons for their occurrence, describe how they were detected, and propose ways to prevent or lessen them.

**Methods:**

Following the PRISMA (Preferred Reporting Items for Systematic Reviews and Meta-Analyses) guidelines, a systematic review was performed to examine the unintended effects of health interventions that use mobile technology.

**Results:**

A total of 15 papers were included in the review. An ecological typology of mHealth intervention unintended effects (mHUE) was developed, which includes 26 distinct effects (eg, silencing and boomerang). The majority of these unintended effects (n=20) occur at the individual level and span physical or behavioral (n=7), psychological (n=8), cognitive (n=4), and financial (n=1) domains. Three effects occur at the interpersonal level and another 3 at the community or institutional level. Most of the identified effects (n=22) were negative. Potential causes for these effects include the improper use of mHealth technology, poorly designed interventions, the application of unsuitable intervention mechanisms, or a misalignment between the intended outcomes and the sociocultural context. Strategies and recommendations (eg, considering the context such as cultural norms) were suggested to help prevent or reduce the unintended effects.

**Conclusions:**

The unintended effects detailed in the mHUE typology were heterogenous and context-dependent. These effects can influence individuals across different domains and also affect unintended people within the ecological system. As most of the unintended effects are negative, if they are not monitored, mHealth interventions designed to empower participants could paradoxically disempower them (eg, decreasing self-efficacy for disease management, undermining patient control, and engagement). The mHUE typology, together with the proposed recommendations and strategies, can be used as a guide to enhance the planning, design, implementation, and postimplementation evaluation on mHealth interventions. Future research should concentrate on understanding the specific mechanisms behind these unintended effects.

## Introduction

Mobile health (mHealth) interventions use mobile technologies to offer medical and public health support with the goal of improving health outcomes [1]. These interventions are generally well received by participants [2]. Existing literature has documented the effectiveness of these interventions in helping children and adolescents change sedentary behavior and improve physical activity [3], as well as in promoting cancer screening and prevention [4]. They are also beneficial for patients with conditions such as coronary artery disease [5] and for those with chronic pain to relieve pain [6]. Furthermore, they can help improve the quality of life for patients with cancer [7].

However, mHealth interventions can also lead to unintended effects (eg, harming patients) [8,9]. Unintended effects are outcomes not planned by implementers (eg, clinicians or health care researchers) [10] and could be unexpected and unanticipated, with potentially negative consequences [8]. It is crucial to examine the unintended effects of mHealth interventions for several reasons [11]. First, understanding both unintended and intended effects provides a more thorough and objective view of their impact [12]. Second, a better grasp of the consequences can help lessen potential harm to patients [13,14]. Third, not addressing unintended effects can undermine the effectiveness and sustainability of mHealth interventions [15,16].

Theoretical frameworks for the evaluation of health promotion interventions, such as the RE-AIM (Reach, Effectiveness, Adoption, Implementation, and Maintenance) framework [17], and frameworks for the study of mHealth interventions (eg, the study by Maar et al [9]) have identified unintended effects as a significant component. Systematic review authors have also called for more research and evidence on unintended effects of mHealth interventions [14,18] because of “insufficient assessment of unintended harms” [19]. Although there is a limited number of existing theoretical frameworks and literature reviews [20-22] on the unintended effects on technology in general [11] and on health IT [8,10], they did not focus on mHealth interventions specifically. For instance, the reviews of unintended consequences associated with health IT [10,23] mainly focused on the adverse effects (eg, physician burnout and unfavorable workflow) of the implementation of electronic health records with health care providers as intended users. However, patients are also important stakeholders of health IT and mHealth interventions. A systematic review that includes all relevant stakeholders (eg, health care providers and patients) would provide a more comprehensive picture of the unintended effects.

Moreover, the existing reviews were published a few years ago and thus might not catch the most recent evidence. In addition, existing relevant reviews have provided useful taxonomy of risks and adverse effects of digital technology, which includes mHealth tools [24]. Their typology [24] included side effects, such as impairment of health and undesirable behavioral adaptation. However, more research is needed on why the effects happen, how these effects are detected, and how to prevent them from happening or minimize the impact in future studies [10]. Therefore, given the importance of the unintended effects and the gap in the existing literature, we propose to conduct a systematic review in this study to examine the unintended effects of mHealth interventions among relevant stakeholders.

The purpose of the systematic review is not to undermine the role of mHealth technologies in health interventions but to come up with a typology of the unintended effects of mHealth interventions and to better understand and manage the unintended effects of mHealth interventions. A typology is useful in that it presents the scattered cases of unintended effects in a systematic and meaningful way [12]. We used the ecological model by McLeroy et al [25] as a framework guide of our study, because we would like to include all the relevant stakeholders involved in the mHealth interventions, such as individual patients or health care providers as users of the mHealth tools, and their environment or the system they are in, such as family, organization, community, cultural context, or society. With the application of this framework, we will be able to not only identify unintended effects that happen at an individual level but also capture those that happen at interpersonal or community levels. The aims of this systematic review are to systematically identify the unintended effects of mHealth interventions and develop a typology, explore the reasons for their occurrence, explain how they were detected, and suggest how to avoid or mitigate them.

The following research questions (RQs) are proposed to guide the systematic review: RQ 1: What are the unintended effects identified in mHealth interventions? RQ 2: Why did the unintended effects happen, or what were the potential causes in mHealth interventions? RQ 3: How were the unintended effects detected in mHealth interventions? Specifically, what research methods were used among the studies in which the unintended effects were detected? RQ 4: How can the unintended effects be prevented or mitigated in future mHealth interventions?

## Methods

### Overview

This systematic review investigated the unintended effects of health interventions that involve mobile technology. The review was conducted in accordance with the PRISMA (Preferred Reporting Items for Systematic Reviews and Meta-Analyses) 2020 guidelines [26] (the PRISMA checklist is provided in Checklist 1). After a systematic literature search, a content analysis [27] of the selected studies was performed to gain a more profound understanding of the unintended effects.

### Search Strategy

The systematic search was conducted using the following databases: PubMed, Embase, CINAHL, Communication and Mass Media Complete, and PsycINFO on April 29, 2023. A combination of the search terms was used, with some keywords about mHealth technology and some other keywords about unintended effects. See Multimedia Appendix 1 for the search strategies and the justification based on our previous study [28].

### Study Selection

We intended to include both quantitative and qualitative literature in the systematic review because we would like to examine the issue from multiple perspectives. More specifically, quantitative research (eg, intervention trials) can identify the effects of interventions from a statistical standpoint, whereas qualitative studies (eg, interviews or focus groups) can explore each participant’s views and experiences [29,30]. Mixed methods studies were also included. A meta-analysis was deemed inappropriate because the studies reviewed were heterogenous [30].

The inclusion criteria and exclusion criteria were described in this section. Studies were included if they met the following criteria: focusing on testing the effects of mobile technology for the purpose of healthcare while also mentioning unintended effects, or examining the participants’ perspectives of the mHealth intervention and mentioning unintended effects. Papers were excluded if they had one or more of the following characteristics: (1) not mHealth interventions, or mHealth intervention studies without mentioning unintended effects, (2) study protocols without results, (3) studies focusing exclusively on mHealth app design and development, (4) theoretical papers or reviews, (5) papers not written in English, (6) not available in full text, or (7) not published in peer-reviewed journals or conferences. Based on the inclusion and exclusion criteria, 2 researchers handled the study selection process, with disagreement resolved through rounds of discussions. Covidence [31] was used to manage the review process. A total number of 2687 papers were identified through database search. The final number of studies included was 15.

### Quality and Risk of Bias Assessment and Data Analysis

The following risk of bias evaluation tools were used in the study, because they have been applied to previously published systematic reviews [28,32]: Cochrane Collaboration’s Risk of Bias Tool for randomized control trials [33], Quality Assessment Tool for Observational Cohort and Cross-Sectional Studies and Quality Assessment Tool for Before-After (Pre-Post) Studies With No Control Group developed by National Institutes of Health [34], and Critical Appraisal Skills Programme for Qualitative Studies [35]. For mixed methods studies, we used 2 tools to evaluate the quantitative and qualitative methods, respectively. First, 2 coders (AS and XC) worked independently to evaluate the quality of the included studies. Then, they compared their results to identify the disagreements that were resolved later after rounds of discussions and a consultation with a third researcher (WC). The results of the evaluations were included in Multimedia Appendices 2-5 [15,33-49]. Overall, for randomized trials, we noted some insufficient reporting in terms of random sequence generation, allocation concealment, blinding of participants and personnel, and incomplete outcome data. For qualitative studies, the adequate consideration of the relationship between research and participants was often not reported.

For the content analysis of the systematic review, 2 coders (WC and XC) independently coded the 15 studies on the characteristics of the studies (eg, study cite and mobile technology used) and key information related to the unintended effects (eg, why they happened). The coding was based on the information reported in the reviewed papers. If, in a paper, no relevant information was provided, or the description was unclear or too general, we coded it as “unknown.” Full agreement between the 2 coders was reached after rounds of discussions.

## Results

### Characteristics of the Studies Reviewed

**Figure 1.**
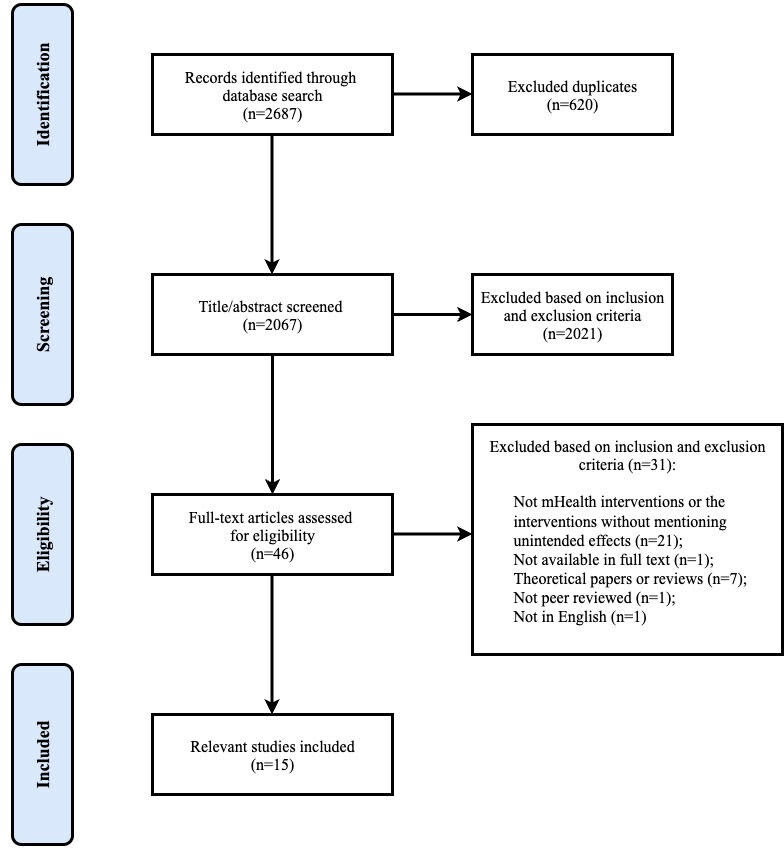
PRISMA (Preferred Reporting Items for Systematic Reviews and Meta-Analyses) chart.

A total of 15 studies were included in the review (Figure 1 shows the PRISMA chart), primarily conducted in Africa [15,36-39] and the United States [40-43]. Five [36,40,41,43,44] were quantitative studies and 7 [15,38,39,42,45-47] were qualitative studies. Three [37,48,49] used mixed methods design. Sample sizes varied greatly, from 6 patient-physician consultations [46] to 1488 participants [44].

The mHealth technology used included clinical decision support systems (eg, Amoakoh and colleagues [36]), iPods used by operating room nurses for their work (eg, Sergeeva and colleagues [45]), computerized physician order entry (eg, Strom and colleagues [43]), mHealth apps (eg, Rudin and colleagues [42]), and smartphone-based information system (eg, Steege and colleagues [39]). The purposes of the mHealth technology ranged from providing clinical decision support (eg, Amoakoh and colleagues [36]) to supporting disease self-management (eg, Rodrigues and colleagues [47]) and helping with symptom monitoring (eg, Rudin and colleagues [42]). The majority of the studies focused on maternal or neonatal or child health [15,36,38,39,41], and HIV/AIDS [37,47]. Intended users of the mHealth technology included both health care workers, such as frontline health workers (eg, Amoakoh and colleagues [36]), and patients, such as maternity patients (eg, Ledford and colleagues [41]).

Six out of 15 studies used theories to explain the unintended effects, such as self-determination theory [50] used in 2 studies [40,41], and Michel Foucault’s [51,52] concept of power and power technology used in 1 study [46]. See Table 1 for more details.

**Table 1.** Characteristics of the studies.

Study	Country /region	Study design	Sample size	Types of mHealth tech	Purposes of mHealth tech	Health conditions or disease studied	Intended users	Theory used
Amoakoh et al [36]	Ghana	Quantitative	8 intervention clusters; 8 control clusters	mHealth^a^ clinical decision-making support intervention	Support clinical decision-making; provide quick and easy access to emergency maternal and neonatal health protocols to frontline health workers	Maternal or neonatal or child health	Frontline health workers	N/A^b^
Austin and Kwapisz [40]	United States	Quantitative	159 participants	Motivational apps	Help people achieve behavioral goals	N/A	University students	Self-determination theory
Chang et al [37]	Uganda	Mixed methods design	20 participants for interviews; 6 focus groups (specific number unknown); 27 for surveys	mHealth intervention; smartphone application	Empower existing community health workers; improve communication; streamline data collection; improve clinical decisions; and receive alerts	HIV/AIDS	Community health workers and clinic staff	N/A
Duclos et al [38]	Burkina Faso	Qualitative	187 participants	mHealth intervention	Increase maternal care access	Maternal or neonatal or child health	Godmothers	N/A
Ledford et al [41]	United States	Quantitative	205 participants	Mobile app	Provide patient education	Maternal or neonatal or child health	Maternity patients	Self-determination theory
Pedersen et al [44]	Denmark	Quantitative	1488 participants	Text messaging via mobile phones’ SMS	Prompt healthier eating behaviors	Healthy eating	Adolescents	Social cognitive theory
Reiss et al [48]	Bangladesh	Mixed methods design	969 participants	An interactive voice message intervention delivered via mobile phone	Support post-MR^c^ contraceptive use	Menstrual regulation	Women who had undergone menstrual regulation	COM-B^d^ model of behavior change for mobile phone intervention
Rodrigues et al [47]	India	Qualitative	16 participants	An automated interactive voice response call; a neutral picture short messaging service (SMS)	Support antiretroviral treatment adherence	HIV/AIDS	Patients with HIV receiving antiretroviral treatment	N/A
Rudin et al [42]	United States	Qualitative	16 participants	mHealth apps	Help with asthma symptom monitoring	Asthma	Patients with asthma	Health belief model
Sergeeva et al [45]	The Netherlands	Qualitative	52 participants	iPods	Improve OR^e^ nurses’ access to information	N/A	Operating room nurses or assistants	Health belief model
Stampe et al [46]	Denmark	Qualitative	6 patient-physician consultations	How-R-you app	Empower patients’ self-management activities; help physicians gain a better understanding of patients’ everyday lives with chronic diseases and the symptoms they experience.	Juvenile idiopathic arthritis	Children and adolescents diagnosed with juvenile idiopathic arthritis	Michel Foucault’s concept of power and power technology
Steege et al [39]	Ethiopia	Qualitative	19 in-depth interviews; 8 focus groups	Smartphone-based information system	Help health extension workers to input data on expectant mothers and TB^f^; prompt HEWs^g^ to follow up on expectant mothers’ due dates and sputum examination for TB symptomatic cases.	Maternal or neonatal or child health; tuberculosis	Health extension workers	McLeroy’s socioecological model
Strom et al [43]	United States	Quantitative	1971 participants	Computerized physician order entry	Alert significant drug-drug interactions; improve prescribing habits	Warfarin and trimethoprim-sulfamethoxazole; drug interaction	Clinicians	N/A
Udenigwe et al [15]	Nigeria	Qualitative	66 participants	SMS text messaging	Increase women’s use of health care facilities for maternal, newborn, and child health in rural Edo	Maternal or neonatal or child health	Pregnant women	Various gender frameworks
Van Olmen et al [49]	Democratic Republic of Congo, Cambodia, and the Philippines	Mixed methods design	1470 participants	mHealth intervention with SMS text messaging	Help with diabetes self-management by SMS text messaging	Diabetes	Patients with diabetes	Intervention theory: Theory of Planned Behavior

a mHealth: mobile health.

b N/A: not applicable.

c MR: menstrual regulation.

d COM-B: capability, opportunity, motivation, and behavior.

e OR: operating room.

f TB: tuberculosis.

g HEWs: health extension workers.

### Unintended Effects Reported in mHealth Interventions

**Figure 2.**
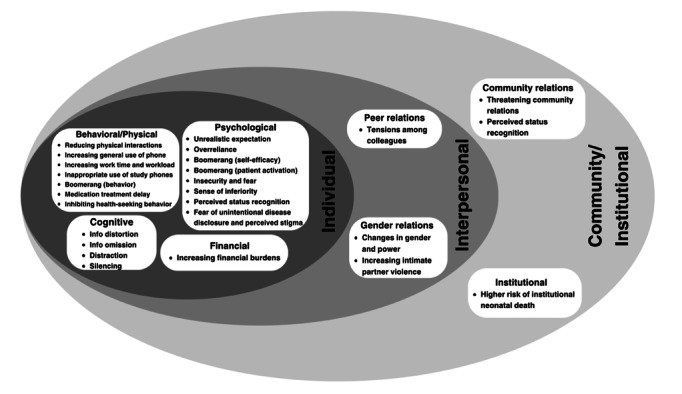
An ecological typology for mHealth intervention unintended effects.

RQ1 asked about what unintended effects have been identified in the studies. And one goal of the study is to develop a typology of the unintended effects based on the ecological model [25]. A total number of 26 unintended effects were identified. In Table 2, we described each unintended effect in detail and also provided the potential cause for each unintended effect. For the wording of the names of the unintended effects, some directly came from the original studies (eg, intimate partner violence [IPV] [48]) if they specifically pointed out the unintended effects; some were analyzed and interpreted by the authors based on their published papers included in our review (eg, boomerang [40], especially if they just pointed out the issue without providing a term for the effect. Similarly, causality was analyzed and interpreted by the authors based on their published papers included in our review. We used the ecological framework [25] to organize the identified unintended effects into different levels and came up with an ecological typology for mHealth intervention unintended effects (mHUE), as shown in Figure 2. Most (n=20) unintended effects happened at the individual level, such as insecurity and fear. The individual-level effects can be further divided into 5 categories: physical or behavioral (n=7; eg, reducing physical interactions), psychological (n=8; eg, overreliance), cognitive (n=4; eg, silencing), and financial (n=1; eg, increasing financial burdens) domains. Some effects happened at the interpersonal level (n=3), influencing peer relations (eg, tensions among colleagues) and gender relations (eg, increasing IPV). Effects can also happen at the community or institutional level (n=3; eg, perceived status recognition by the community).

**Table 2.** Unintended effects of mHealth interventions and the potential causes.

Unintended effects	Definitions	Potential causes	Examples
Adopting study phone for personal use	Some health extension workers who were provided with the study phones considered the phones their personal property and used them for personal purposes (eg, making phone calls).	Intervention participants’ perception: The users considered the study phone as their personal property.	Steege et al [39]
Boomerang (behavioral)	The response or effect experienced by the intervention participants is opposite to the intended or expected effects of the mHealth^a^ interventions. It can happen at the behavioral level (eg, performing target activities).	Intervention: Specific design features of the mHealth intervention (eg, daily reminders) promote external motivation for performing a behavior and thus participants’ internal motivation has been undermined. This violates the basic tenets of self-determination theory.	Austin and Kwapisz [40]
Boomerang (psychological: eg, self-efficacy and patient activation)	The response or effect experienced by the intervention participants is opposite to the intended or expected effects of the mHealth interventions. It can happen at the psychological level (eg, self-efficacy of healthy behaviors or patient activation).	Intervention: Certain features of the mHealth intervention (eg, daily text messages) have reminded participants of their failure to perform a challenging task, making the participants feel the lack of confidence in their ability to achieve the goal [44]. The reason for lowering patient activation could be explained using the self-determination theory that particular design features of the mHealth intervention (eg, health information provided in the app) make users feel not internally motivated to seek health information in an electronic modality [41].	Ledford et al [41]; Pedersen et al [44]
Distraction	mHealth users get distracted by the device by focusing on non–work-related features of the device or system.	Intervention participants’ behaviors: Using the mHealth system or device for non–work-related purposes.	Sergeeva et al [45]
Fear of unintentional disease disclosure and the stigma	Patient users of the mHealth intervention fear that their disease status (eg, HIV infection) gets disclosed and fear that they will be stigmatized because of their disease status.	Intervention: According to study participants, "frequent calls might increase the risk of unintentional disclosure of their HIV status as the likelihood of someone else receiving the call while the phone is unattended cumulates with the number of calls received.”	Rodrigues et al [47]
Higher risk of institutional neonatal death	The higher risk of institutional neonatal death observed in intervention clusters, and the purpose of the mHealth intervention is clinical decision-making support aiming at improving neonatal mortality.	Intervention: May be “problems with birth and death registration, unmeasured and unadjusted confounding, and unintended use of the intervention”	Amoakoh et al [36]
Increasing financial burdens	The health extension workers have to pay extra money to use the study phone.	Intervention: The running costs (eg, airtime charges) of the phone and the cost of the phone if it is stolen or lost.	Steege et al [39]
Increasing general use of phone	Participants use their phones more frequently than before, not only for disease management purposes but also for general purposes.	Technology: It could be that phones have other functions, and using them for disease management would increase their access to phones, and thus providing them with more opportunities to use them for other purposes. (The reason was the authors’ interpretation, because the reason was not mentioned in the paper reviewed).	Van Olmen et al [49]
Increasing intimate partner violence	The female participants experience violence (eg, beating) from their husbands or intimate partners.	Intervention and cultural context: Some female participants did not seek their husbands’ permission before making a decision (eg, participating in the mHealth intervention), and in those cultures, women need to obtain consent from their husbands before making a decision.	Reiss et al [48]
Increasing work time and workload	The mHealth system might increase the work time and workload of health care workers (eg, health extension workers).	Intervention: The intervention had limited capacity and the “health extension workers had to spend extra time in putting data, as paper-based reporting was still required.”	Steege et al [39]
Information distortion	The intended users of mHealth technology or mHealth interventions will misrepresent or misinterpret the information presented in mHealth technology or mHealth interventions.	Intervention participants: The intended users did not know how to operate the phone properly or did not have the digital skills to operate the phone.	Chang et al [37]
Information omission	Some information presented in the mHealth technology or mHealth intervention will be missed, and the intended users of the mHealth technology will not get a complete picture of the information.	Intervention participants: The intended users did not know how to operate the phone properly or did not have the digital skills to operate the phone.	Chang et al [37]
Inhibiting health-seeking behavior	Patients who are overreliant on the mHealth system will not actively seek health care and wait for health care providers to call them.	Intervention participants: Overreliance on the mHealth system.	Duclos et al [38]
Insecurity and fear	The feeling of not being safe and the experience of fear of walking with the phone, especially at night, as well as the fear of phone loss or theft.	Intervention and environmental context: The need to carry study phones with them and the phones are considered as valuables in an environment that is not safe.	Chang et al [37]; Steege et al [39]
Medication treatment delay	Patients whose prescribing physicians are in the intervention group and who need immediate drug therapy are unable to get the therapy on time.	Intervention: Because the physicians did not prescribe the medications due to the computerized physician order entry alert to prevent a drug interaction.	Strom et al [43]
Negative changes in gender and power relationships	Because women participated in mHealth, their decision-making ability and autonomy have been enhanced (intended positive effect), which threatens men who assume to maintain control over women, in some cultures. This has the potential to increase domestic conflicts.	Intervention and the social cultural context: The increase of women’s status threatens men’s control over women and is against some existing cultural and social norms.	Udenigwe et al [15]
Overreliance	mHealth could disrupt the existing practices (ie, patients taking an active role in contacting and seeing their health care providers). With the ongoing use of mHealth systems, eventually, patients would rely on external factors (eg, the app system or health care workers) to call them to see health care providers.	Intervention: mHealth users got used to the system that emphasizes using external factors rather than internal factors to increase health care access.	Duclos et al [38]; Rudin et al [42]
Perceived status recognition	Health extension workers who had the mobile phone for research purposes perceived that the community members thought that they had a higher status because they had information about the mobile phone, including its purpose.	Intervention context: The perception of the community that a person’s status is linked to technology (eg, smartphones) and its associated skills.	Steege et al [39]
Positive changes in gender and power relationships	The intervention has elevated the female health extension workers’ status at home because the females have gained access to the smartphones and are the ones to manage the study phones. They reported not allowing their husbands or children access.	Technology and intervention: Female participants have gained access to intervention smartphones and thus have gained the power to be the gatekeepers of study mobile phones. The power dynamic of men as mobile phone gatekeepers in some cultures has been inverted.	Steege et al [39]
Reducing physical interactions	People think that technology would solve all the problems and, therefore, reduce face-to-face interactions.	Intervention participants’ perception: The perception that technology can solve all the issues in the mHealth context.	Chang et al [37]
Sense of inferiority	Other health extension workers who were not engaged in the study and were not provided with the study phones, as well as the associated knowledge and skills, felt that they had lower status than those who were provided with the study phones.	The unintended audience’s perception: The perception that people with access to the study phones and their associated knowledge and skills have a higher status than those who do not have the phones and the knowledge and skills.	Steege et al [39]
Silencing	Patients’ voices have been prohibited because the mHealth system instructs them to express themselves in a specific way and does not allow patients to express themselves freely. The patients’ descriptions of the disease may be ignored.	Intervention and technology: The mHealth system “orders patients’ words and presents them in a specific way.”	Stampe et al [46]
Tension among colleagues	The negative interpersonal relationship between the health extension workers who participated in the mHealth intervention and their supervisors who did not.	Government supervisors without smartphone access and the associated skills felt that they could not properly manage extension workers with the technology and skills.	Steege et al [39]
Threatening community relationships	The increase of women’s gender and power status as a result of mHealth intervention participation threatens community relationships.	Intervention and the social cultural context: The benefits (eg, elevating women’s status) and recommendations of the mHealth interventions conflict with the culture’s social norms (ie, men maintaining control over women).	Udenigwe et al [15]
Unrealistic expectation	Some mHealth users have the anticipation of specific tasks that exceed certain limits and are not realistic or achievable within the mHealth context. For instance, community health workers might call clinic staff and ask them to provide a medical treatment solution without them seeing the patient and without them knowing the patient’s medical history.	Intervention participants: Health extension workers might be unfamiliar with the clinic staff’s job characteristics and responsibilities and unaware of the tasks that are not achievable within the mHealth context.	Chang et al [37]

a mHealth: mobile health.

### Positive and Negative Unintended Effects

The review identified 1 neutral effect (ie, increasing general phone use [49]) and 2 positive unintended effects: positive changes in gender and power relationships [39] and perceived status recognition [39]. All the other (n=22) identified effects were negative effects.

### Potential Causes of the Unintended Effects

To answer RQ2, the specific cause for each unintended effect is shown in Table 2. In sum, the unintended effects were due to several factors. It could be related to technology (eg, phone loss or theft [39] and financial burdens [39]), or because of technology not being appropriately used (eg, phones not properly used [37]), or because of mastering the skills related to the technology or intervention (eg, tension among colleagues [39]). It could be because of the intervention participants not being properly trained (eg, inappropriate perceptions [39] or behaviors [45]), the intervention not being appropriately administered [36], or because of the choice of a specific intervention mechanism (eg, patients rely on external factors to motivate them to see their health care providers [38,42]). Moreover, unintended effects could happen because the changes enabled by mHealth interventions did not agree with social and cultural norms (eg, men’s dominant status in the family was threatened [15]). In addition, specific app or intervention features (silencing effect due to app design [46]) and limitations of interventions (the limited intervention capacity [39]) could also be the reasons.

### Detection of the Unintended Effects

RQ3 asked how the unintended effects were detected or what methods were used in the studies that reported the unintended effects. The unintended effects were identified using qualitative research methods such as interviews, focus groups, or ethnographic field studies [15,37-39,45-47,49]. The qualitative methods were used to explore users or participants’ perspectives that were not examined in the quantitative outcomes.

The effects were also detected through quantitative research: cluster-randomized controlled [36], randomized controlled trial [41,48], between-subjects factorial design experiment [40], and pre-post experiment with control group [44]. The unintended effects were discovered because the results were unexpected and were in the opposite direction of the hypotheses. For instance, the patient activation levels of the participants in the mobile app group were hypothesized to be higher than the levels of the participants in the control group. However, the results showed the opposite [41]. In one study, the unintended effects were identified by the research team [42] without specifying the specific detection method. In another study, monthly monitoring was used to discover the unintended effects [43].

It is important to note that one group of researchers [48] shared the specific ways they used to measure the unintended effect of IPV and emphasized that “IPV must be measured using closed questions naming acts of violence” [48]. In their study, physical IPV was measured by 2 means, using a direct question that named specific acts of violence (ie, physical IPV, sexual IPV, and physical violence perpetrated by the participant’s in-laws), and using an open question about positive or negative effects of being in the study, and they were able to detect the IPV through closed questions [48].

### Prevention and Mitigation of Unintended Effects

**Figure 3.**
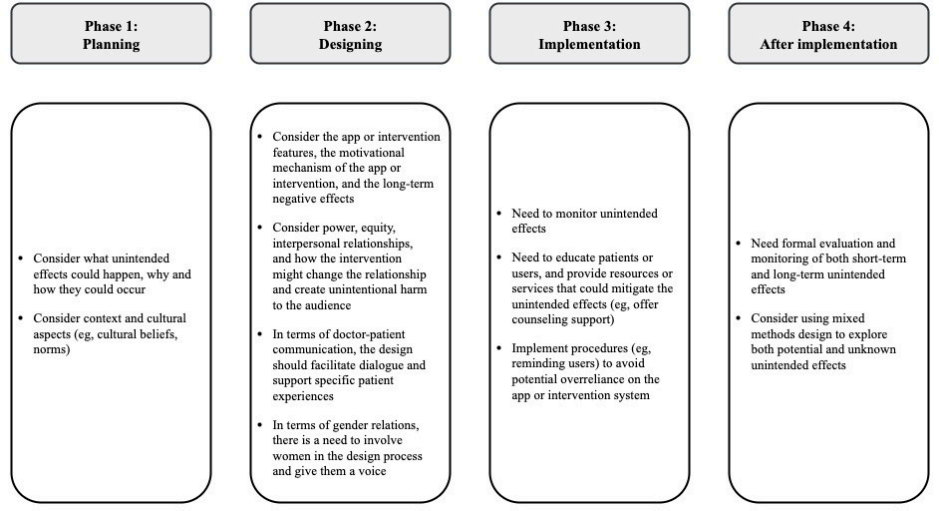
Strategies and recommendations to prevent and mitigate the unintended effects.

RQ4 asked how to prevent or mitigate the unintended effects. The reviewed studies shared their strategies and recommendations (summarized in Figure 3) by naming the factors to be considered at various stages or processes of intervention. When planning interventions, researchers need to consider what unintended consequences (eg, boomerang effect) could happen, and why and how they could occur [44,48]. At the planning stage, researchers should also consider the context [53] of the participants and the cultural aspects (eg, cultural beliefs and norms [54,55]). According to one study [48], “In Bangladesh, not seeking a husband’s permission before making a decision is often reported to warrant wife-beating, and a husband’s lack of approval was also given as a reason for refusal to participate in the study.”

When designing apps or interventions, researchers and app developers may need to consider the app features and their motivating mechanisms. When designing and introducing technology and mHealth interventions, we should consider power, equality, interpersonal relationships, and the influence on unintended audience (eg, intimate partners or husbands of the intervention participants), and how the technology or the intervention might change the relationship and create unintentional harm to the intended audience. Specifically, in doctor-patient communication, the design should facilitate dialogue and support specific patient experiences rather than using a structure to silence patients’ voices [46]. In terms of gender relations, husbands as unintended audiences have significant involvement in maternal health in some cultures, and the mHealth intervention unintentionally generated more conflict. There is a need to involve women in the design and implementation of maternal health programs to give them a voice in determining the programs they need [15].

Before and during implementation, there is the need to educate patients or users and provide resources or services that could mitigate the unintended effects. For instance, ensuring confidentiality [48] and offering counseling support could be a way to overcome HIV status stigma associated with intervention [47]. To avoid potential overreliance on the app, researchers need to remind patients that the app will not make decisions for them, and researchers need to include reminders in apps to inform them of the option of calling their doctor or going to the emergency room [42]. Necessary training sessions are needed to train the intervention participants to prevent unintended effects such as information omission, information distortion, and unrealistic expectations [37].

Formal evaluation and monitoring of interventions [43,54] for potential unintended effects are strongly encouraged. It is better to use mixed methods design [10] to gain deeper understanding and more comprehensive picture of the effects.

## Discussion

### Principal Findings

In this systematic review, we examined 15 papers reporting unintended effects in their research involving mHealth technology and identified 26 heterogeneous unintended effects at individual (n=20), interpersonal (n=3), and organizational or community (n=3) levels. A typology of mHUE has been developed. The potential reasons for the unintended effects include mHealth technology not appropriately used, or intervention not appropriately designed, intervention participants not properly trained, or inappropriate intervention mechanism applied, or the intended intervention outcomes not in agreement with the social and cultural context. The recommendations and strategies (eg, considering the context such as cultural norms) were proposed to help avoid or mitigate the unintended effects. In the following sections, we will deeply analyze the patterns, contradictions, and gaps in this area and elaborate on how negative unintended effects, if left uncontrolled or unmonitored, can negatively influence patient empowerment.

### Unintended Effects on Unintended Audiences

In our review, we identified 26 unintended effects, such as negative changes in gender and power relationships, and overreliance. Similarly, papers examining the information technologies in health care focusing on clinicians found the following unintended effects: more workload for clinicians, changes in communication patterns (eg, decline of vital interaction among care providers), changes in power structure (eg, IT and administration gain power by requiring physicians to comply with requirements), and health care professionals’ overdependence on the technology [20,56].

However, our review also found that unintended effects could also happen to the unintended audience of the mHealth interventions, which could have negative impact on interpersonal and community relationships. These unintended audiences include patients involved in the mHealth clinical decision support system for clinicians (eg, higher risk of neonatal death observed in the mHealth clinical decision-making support intervention arms [36]), husbands or intimate partners of the intervention participants (eg, the female participants who did not seek the approval of their husbands or intimate partners to participate in the study experienced violence from their husbands or intimate partners [48]), eligible potential participants but not in the intervention arm (eg, other health extension workers who were not engaged in the study experienced a sense of inferiority [39], the supervisors of the health extension workers who felt the pressure to manage those who were engaged in the intervention [39]), or the community leaders and members who perceived that the change in increasing gender and power among women would threaten community relationships [15].

### Unintended Effects and the Underlying Mechanisms

The underlying mechanism of some unintended effects (including boomerang effect at behavioral and psychological levels, inhibiting health-seeking behavior, and overreliance) can be explained using self-determination theory [50], which highlights the important role of competence and internal motivation in behavior change [57]. However, specific mHealth intervention features (eg, daily text messages and daily reminders) focus on facilitating participants’ engagement with the technology, rather than on supporting competence, or self-efficacy of healthy behaviors or patient activation, rather than on facilitating internalizing and valuing the intervention goals (eg, health behavior change) to promote intrinsic motivation [58]. Overemphasizing external motivations could explain why the unintended effects happened.

Some unintended effects (eg, increasing IPV, negative changes in gender, and power relationships) could be explained using gender and power norms in societies. In patriarchal societies, females’ health decisions are often made by their husbands or male partners. Per Foucault’s concept of technologies of power [51], these tools, designed to help females’ autonomy and independence, redistribute access to knowledge and decision-making and shift power in a way that threatens male authority. Therefore, mHealth tools that aim to promote female autonomy can challenge traditional gender roles, triggering interpersonal conflict or IPV.

Digital literacy, the competencies and skills needed for effectively using a mHealth system [59], can be used to explain the unintended effects of information distortion and information omission. Studies have demonstrated digital literacy effects on both patient expertise and mHealth app usability [60]. More studies are needed to explore the relationships among these factors. For instance, lower digital literacy could lead to more information distortion and information omission, which then negatively impacts patient expertise and app usability.

### Unintended Effects and Intervention Context

Similar to the mixed intended effects of mHealth interventions on gender and power relationships found in the review [61], in our review, we observed some mixed results of the unintended effects in this domain. One study [39] reported positive changes (eg, female participants’ higher status at home and in a community) in gender and power relationships, while other studies [15,48] reported negative changes (eg, domestic conflicts or violence experienced by female participants). This raises the question of whether or not access to technology and intervention will increase women’s status and power at home or in a community. A closer examination of the 3 studies revealed similarities and differences in terms of social and cultural contexts. All of them involved women as the intended users of the mHealth technology in the male dominance hierarchy context, specifically, with “patriarchal norms where men are the primary keepers of technology” [39], or of “entrenched systems of power through which men control women’s access to resources and their reproductive and social lives” [15], or where “not seeking a husband’s permission before making a decision is often reported to warrant wife-beating” [48]. When it comes to maternal health care and women’s reproductive health, gender and power inequalities and dynamics should be taken into consideration in the mHealth interventions [15,48]. The reason why the study by Steege et al [39] reported positive social status and agency changes among women was because they were health extension workers who were government employees with superior social status already and thus were able to invert “the traditional patriarchal norms” [39] with the help of the mHealth intervention.

Previous studies have highlighted the importance of considering social cultural factors in the design (eg, language, layout, and interface) of mHealth interventions from the perspective of increasing access, use, and long-term engagement [62]. Our review also echoes the importance of social cultural context but from the perspective of avoiding or mitigating unintended effects in mHealth interventions.

### Unintended Effects and (Dis)Empowerment

Most of the unintended effects discovered in our study were negative. They can negatively influence various domains (eg, cognition and behavior) of an individual. As mentioned previously, they also negatively influence interpersonal (eg, doctor-patient, husband-wife, and colleagues) and community relationships. These findings further emphasize the need to systematically monitor the unintended effects in future mHealth interventions in order to avoid potential disempowerment or even harm to participants.

Those mHealth interventions, if not addressed, have the potential to disempower users or participants, which is in the opposite of the intended goal of many mHealth interventions. The framework [63] with 6 dimensions of empowerment could help further explain in what specific aspects (dis)empowerment could happen: control (ie, the ability to make health care decisions and to decide levels of health care engagement), psychological coping (the state of one’s psychological process of adapting to the negative changes due to one’s health status or disease), self-efficacy (one’s cognitive and physical capabilities for self-care or disease self-management), understanding (one’s capacity to apply information and knowledge for health care and disease management), legitimacy (one’s perception that the care received from a health care professional or from a health care system is fair as well as one’s trust in the professional or health care system), and support (the quality or quantity of one’s perceived available support from the care provider or the nonmedical supporting environment). Bravo and colleagues [64] also identified similar indicators of patient empowerment, including self-efficacy, knowledge, skills, perceived personal control, feeling respected, making informed decisions, and taking an active role in health care.

Based on the framework [63], we argue that at least 5 of the 6 dimensions of empowerment could be impacted due to the unintended effects: control, psychological coping, self-efficacy, understanding, and support. Unintended effects such as information distortion and omission will negatively influence users’ understanding of their health conditions and disease self-management, which subsequently influences their control and the ability to make informed and appropriate health care decisions. Unintended effects such as overreliance on mHealth apps and inhibiting health-seeking behavior will influence control. Relying too much on external factors, one will gradually lose the ability to take the initiative to engage in health care actively. Unintended effects such as silencing patients’ voices will also influence control because the app features limit patients’ expressions and leave patients’ experiences and voices unheard. Unintended effects such as the boomerang effect will hurt self-efficacy because users or patients have been motivated externally by some app features and will lose internal motivation to perform a health behavior. Unintended effects that include negative changes in interpersonal relationships (eg, gender, colleague relations, and doctor-patient communication) may influence support. Because of the negative interpersonal relationships in one’s environment, one is less likely to receive support from spouses, coworkers, or doctors. Unintended effects such as fear of unintentional disease disclosure and the stigma are related to the domain of psychological coping because one has to use psychological coping strategies to deal with the intervention-related stigma that could have been prevented.

### Gaps in Literature and Future Research

The review also noted significant gaps in the literature. Our quality assessment results revealed insufficient reporting of key methodological elements, including random sequence generation, allocation concealment, and participant blinding in quantitative studies. In qualitative studies, many failed to report whether the nature of the researcher-participant relationship was sufficiently considered in the study. These gaps may impact our subsequent interpretation of unintended effects. For instance, it has been argued that the lack of blinding may have introduced bias in how participants perceived or reported their experiences [65]. In addition, participants’ disclosure also depends in part on their relationship with researchers [66]. As such, participants might have underreported unintended effects due to reasons such as fear of judgment, social desirability bias, or lack of trust in the research team. This limitation underscores the need for transparent reporting and trust-building strategies in future mHealth intervention research, especially when exploring unintended consequences.

Participant dropout or low engagement could be due to the experience of unintended effects [14]. None of the studies reviewed had specifically examined or reported the potential unintended effects among the participants who did not engage much or dropped out of an mHealth intervention. Therefore, the perspectives of the specific patient group were not reflected in the results of our study.

Discerning long- and short-term effects is very important as some effects could be stronger as time lapses [12]. However, most of the studies did not specify whether the unintended effects were for the short or long term. The duration of the effects can depend on various factors (eg, the duration of the intervention, individual characteristics, and the context). It is strongly encouraged that future studies should consider reporting the duration of the unintended effects.

In our review, we summarized the potential reasons for the unintended effects and identified multiple crucial factors to be considered when planning mHealth interventions such as culture, power, and gender relations. Although some existing theories (eg, self-determination theory [67]) can be used to explain the mechanism why some unintended effects (eg, overreliance) happen, we still lack the knowledge of other specific mechanisms to explain the unintended effects. For instance, under what specific situation will the silencing effect happen? Who is more likely to experience the silencing effect? What patient factors (eg, disease self-management self-efficacy) or physician factors (eg, patient-centered communication) will interact with technology factors (eg, mHealth app feature) to influence the silencing effect? Future research should explore the potential mechanism or theoretical framework to better understand unintended effects [10].

In terms of how to avoid future unintended effects, a paper [53] on gender dynamics and digital health also emphasized the ongoing need to monitor the dynamic interactions between gender, power, and digital health and asked the key questions to avoid potential unintended consequences in future. For instance, do marginalized gender groups (eg, women) “have sufficient literacy, autonomy, and ICT access to effectively use digital health?” “How will use of digital health impact and change existing gender power dynamics and relationships among key stakeholders?” [53]. Both that study and our results have indicated that emphasis should also be given to interpersonal effects and even effects at the community level. Implementing digital technology can affect many levels, such as individual or personal, microsystem or interpersonal, mesosystem or organizational, ecosystem or interorganizational, and macrosystem or societal [24]. Societal effects have not been included in our typology because the papers included in our review did not report the unintended effects. Therefore, future research should also explore the unintended effects at the societal level. Future research should also focus on exploring and testing the solutions that prevent or minimize the impact of unintended consequences [10].

Given the implications of the results (eg, the involvement of unintended audience and the unique context of each intervention) and aforementioned gaps (eg, insufficient reporting of key methodological elements and unclear mechanism) in existing studies, we do not know enough to intervene. Although some unintended effects have clear causes, more research is needed to test the effectiveness of the strategies and specific approaches to prevent or mitigate the unintended effects. Of course, there could be other unexpected unintended effects down the road, but with the help of our mHUE typology, previously unexpected unintended effects will be considered in the intervention planning stage to minimize potential harm and to maximize the benefits of mHealth interventions. mHUE will be updated as more interventions start to monitor the unintended effects and report more details of the study in the future.

### Limitations

In addition to the aforementioned gaps in the literature, our study has several limitations. First, we mentioned only some factors related to unintended effects, such as culture and power. However, it is quite possible that other factors (eg, health literacy) beyond what is listed in this review can also contribute to the unintended effects. Second, some findings of this review were context specific and were based on qualitative studies or mixed methods studies involving qualitative studies and therefore may not be gendered to other contexts [61]. Third, due to the inclusion criteria (eg, journal papers, in English, and available in full text) of the systematic research, we might miss papers focusing on the unintended effects but did not meet the criteria.

### Conclusions

In this systematic review examining the unintended effects of mHealth interventions, we developed a typology (ie, mHUE) of 26 heterogenous unintended effects at individual, interpersonal, and community levels. Individual effects can happen in various domains, such as cognitive, psychological, and behavioral ones. We also summarized the potential reasons for the unintended effects and synthesized recommendations and strategies to help avoid or mitigate the unintended effects. Most effects were negative and can disempower the very individual, in various domains (eg, cognitive and behavior), they are meant to help. The unintended effects could also happen to unintended audiences, and the effects should be examined within certain social cultural contexts. Although there is some exploration of the underlying mechanisms to explain the unintended effects, much more research is needed to systematically monitor unintended effects, explore the mechanisms of unintended effects, and test the effectiveness of the proposed prevention or mitigation strategies. The developed typology (mHUE) and the suggested strategies can guide researchers and developers in creating more effective and safer mHealth interventions. More research is needed to explore the specific mechanisms of these effects and to test strategies for their prevention.

## Supplementary material

10.2196/68909 Multimedia Appendix 1 Systematic review search strategies and the justification.

10.2196/68909 Multimedia Appendix 2 Risk-of-bias assessment for randomized controlled trials.

10.2196/68909 Multimedia Appendix 3 Risk-of-bias assessment for pre-post study without a control group.

10.2196/68909 Multimedia Appendix 4 Risk-of-bias assessment for qualitative studies.

10.2196/68909 Multimedia Appendix 5 Risk-of-bias assessment for observational and cross-sectional studies.

10.2196/68909 Checklist 1 PRISMA (Preferred Reporting Items for Systematic Reviews and Meta-Analyses) checklist.

## Abbreviations

IPV: intimate partner violence
mHealth: mobile health
mHUE: mHealth intervention unintended effect
PRISMA: Preferred Reporting Items for Systematic Reviews and Meta-Analyses
RE-AIM: Reach, Effectiveness, Adoption, Implementation, and Maintenance
RQ: research question
