# Supplementary material for: Planning for the Unexpected and Unintended Effects of mHealth Interventions: Systematic Review
Source: J Med Internet Res. 2025 Aug 7;27:e68909. doi: 10.2196/68909 (PMC12331364; doi:10.2196/68909)
Supplement: Multimedia Appendix 1 [file jmir-v27-e68909-s001.docx]

Appendix 1. Systematic review search strategies and the justification.

| Databases | Keywords/Search Strategy | Justification | Results |
| --- | --- | --- | --- |
| Pubmed | (unintended[Text Word] OR unanticipated[Text Word] OR undesired[Text Word] OR unexpected[Text Word] OR unforeseen[Text Word]) AND (effect[Text Word] OR effects[Text Word] OR consequence[Text Word] OR consequences[Text Word] OR influence[Text Word]) AND (mHealth[Text Word] OR m-health[Text Word] OR mobile[Text Word] OR mobile app[Text Word] OR mobile apps[Text Word] OR mobile application[Text Word] OR mobile applications[Text Word] OR smart phone[Text Word] OR smartphone[Text Word]) | We used the search strategy because of the following reasons: 1. The search strategy was used to identify relevant empirical studies which reported unintended effects.  2. The keywords selection were based on previously published related systematic reviews. 3. The strategy was reviewed and approved by someone with mHealth intervention research background. | 308 |
| Embase | (unintended OR unanticipated OR undesired OR unexpected OR unforeseen) AND (effect OR effects OR consequence OR consequences OR influence) AND (mHealth OR m-health OR mobile OR mobile app OR mobile apps OR mobile application OR mobile applications OR smart phone OR smartphone) |  | 668 |
| CINAHL | TX (unintended OR unanticipated OR undesired OR unexpected OR unforeseen) AND (effect OR effects OR consequence OR consequences OR influence) AND TX (effect OR effects OR consequence OR consequences OR influence) AND TX (mHealth OR m-health OR mobile OR mobile app OR mobile apps OR mobile application OR mobile applications OR smart phone OR smartphone) |  | 1496 |
| Communication and Mass Media Complete | TX (unintended OR unanticipated OR undesired OR unexpected OR unforeseen) AND (effect OR effects OR consequence OR consequences OR influence) AND TX (effect OR effects OR consequence OR consequences OR influence) AND TX (mHealth OR m-health OR mobile OR mobile app OR mobile apps OR mobile application OR mobile applications OR smart phone OR smartphone) |  | 115 |
| PsycINFO | TX (unintended OR unanticipated OR undesired OR unexpected OR unforeseen) AND (effect OR effects OR consequence OR consequences OR influence) AND TX (effect OR effects OR consequence OR consequences OR influence) AND TX (mHealth OR m-health OR mobile OR mobile app OR mobile apps OR mobile application OR mobile applications OR smart phone OR smartphone) |  | 100 |
