# Supplementary material for: Planning for the Unexpected and Unintended Effects of mHealth Interventions: Systematic Review
Source: J Med Internet Res. 2025 Aug 7;27:e68909. doi: 10.2196/68909 (PMC12331364; doi:10.2196/68909)
Supplement: Multimedia Appendix 2 [file jmir-v27-e68909-s002.docx]

Appendix 2. Risk-of-bias assessment for randomized controlled trials using Cochrane Collaboration’s Risk of Bias Tool for randomized controlled trials [33].

| Randomized controlled trial | 1. Random sequence generation | 2. Allocation concealment | 3. Blinding of participants and personnel | 4. Blinding of outcome assessment | 5. Incomplete outcome data | 6. Selective reporting | 7. Other bias |
| --- | --- | --- | --- | --- | --- | --- | --- |
| Amoakoh et al (2019) [36] | √ | √ | x | √ | √ | √ | √ |
| Ledford et al (2018) [41] | ? | ? | √ | √ | √ | √ | x |
| Olmen et al (2017) [49], quantitative part | ? | ? | ? | ? | √ | √ | x |
| Pedersen et al (2016) [44] | ? | ? | √ | √ | ? | √ | √ |
| Reiss et al (2019) [48], quantitative part | √ | √ | √ | x | √ | √ | √ |
| Strom et al (2010) [43] | √ | x | ? | ? | √ | √ | √ |
| Note: √: low risk of bias; x: High risk of bias; ?: unclear risk of bias. | | | | | | | |
