# Supplementary material for: Planning for the Unexpected and Unintended Effects of mHealth Interventions: Systematic Review
Source: J Med Internet Res. 2025 Aug 7;27:e68909. doi: 10.2196/68909 (PMC12331364; doi:10.2196/68909)
Supplement: Multimedia Appendix 4 [file jmir-v27-e68909-s004.docx]

Appendix 4. Risk-of-bias assessment for qualitative studies, using the tool of critical appraisal skills program for qualitative studies [35].

| Qualitative Study | | Chang et al (2013) [37] (qualitative part) | Duclos et al (2017) [38] | Olmen et al (2017) [49] (qualitative part) | Reiss et al (2019) [48] (qualitative part) | Rodrigues et al (2015) [47] | Rudin et al (2017) [42] | Sergeeva et al (2016) [45] | Stampe et al (2021) [46] | Steege et al (2018) [39] | Udenigwe et al (2022) [15] |
| --- | --- | --- | --- | --- | --- | --- | --- | --- | --- | --- | --- |
| 1. Was there a clear statement of the aims of the research? | √ | | √ | √ | √ | √ | √ | √ | √ | √ | √ |
| 2. Is a qualitative methodology appropriate? (broad sense) | √ | | √ | √ | √ | √ | √ | √ | √ | √ | √ |
| 3. Was the research design appropriate to address the aims of the research? | √ | | √ | √ | √ | √ | √ | √ | √ | √ | √ |
| 4. Was the recruitment strategy appropriate to the aims of the research? | √ | | √ | NR | √ | √ | √ | NR | x | x | √ |
| 5. Was the data collected in a way that addressed the research issue? | √ | | √ | √ | √ | √ | √ | √ | √ | √ | √ |
| 6. Has the relationship between researcher and participants been adequately considered? | NR | | NR | NR | NR | NR | NR | NR | √ | NR | √ |
| 7. Have ethical issues been taken into consideration? | √ | | √ | NR | √ | √ | NR | NR | √ | √ | √ |
| 8. Was the data analysis sufficiently rigorous? | √ | | √ | √ | NR | √ | √ | √ | √ | √ | √ |
| 9. Is there a clear statement of findings? | √ | | √ | √ | √ | √ | √ | √ | √ | √ | √ |
| 10. How valuable is the research? | √ | | √ | NR | √ | √ | √ | √ | √ | √ | √ |
| Note: √; Yes; x; No; NR; not reported. | | | | | | | | | | | |
